# Supplementary material for: How μ-opioid receptor recognizes fentanyl
Source: Nat Commun. 2021 Feb 12;12:984. doi: 10.1038/s41467-021-21262-9 (PMC7881245; doi:10.1038/s41467-021-21262-9)
Supplement: Supplementary file 1 — Supplementary Information [file 41467_2021_21262_MOESM1_ESM.pdf]

Supplementary Information:

How μ-Opioid Receptor Recognizes Fentanyl

Quynh N. Vo,[†,‡] Paween Mahinthichaichan,[‡] Jana Shen,[*,‡] and Christopher R. Ellis[*,†]

† *Center for Drug Evaluation and Research, United State Food and Drug Administration, Silver Spring, Maryland 20993, United States*

‡ *Department of Pharmaceutical Science, University of Maryland School of Pharmacy, Baltimore, Maryland 21201, United States*

E-mail: jana.shen@rx.umaryland.edu; christopher.ross.ellis@gmail.com

List of Supplementary Tables

1 Simulation steps for apo active mOR 3
2 Simulation steps to relax the docked structure 3
3 Simulation steps to relax the H297 binding mode 4
4 Calculated pK_a's of mOR and fentanyl 4

List of Supplementary Figures

1 Equilibration of the docked fentanyl-mOR structure 5
2 Fentanyl position in the WE-HIE simulation 6
3 Fentanyl position in the WE-HID simulation 7
4 The pK_a of His297 is converged in the replica-exchange CpHMD simulations 8
5 The D147 salt bridge is stable regardless of His297's protonation state . . . 9
6 Aromatic stacking between fentanyl's phenethyl group and Trp293 of mOR . 10
7 Occupancies of the intra- and intermolecular hydrogen bonds 11
8 Fentanyl is pushed away from HIE297 or HIP297 12
9 Trp293 χ_2 angle is different from the BU72-bound mOR structure 13
10 Fentanyl adopts different orientations in the two binding modes 14
11 His297's χ_2 angle is influenced by the protonation state 15
12 Characterization of the equilibrium simulations 16
13 Comparison between fentanyl-mOR contacts and BU72-, DAMGO-, or β-FNA contacts with mOR 17

Supplementary Tables

Supplementary Table 1 Steps in the equilibration simulation of apo active mOR

Step	Ensemble	Time (ns)	BB [a]	mOR Wat[a]	Lipid[b]
1	NVT	0.35	10.0	5.00	5.0 / 2500
2	NVT	0.15	5.00	2.50	5.0 / 200
3	NPT	0.50	5.00	2.50	5.0 / 200
4	NPT	0.50	2.50	1.25	2.0 / 100
5	NPT	1.00	2.50	1.25	1.0 / 100
6	NPT	1.00	2.50	1.25	0.5 / 50
7	NPT	4.00	2.50	1.25	0
8	NPT	60.0	2.50	1.25	0
9	NPT	50.0	0	0	0

[a] Force constant in the harmonic restraint potential for the backbone heavy atoms in kcal/mol/Å^2. [b] Force constant in the position/dihedral restraint potential. The unit is kcal/mol/Å^2 for the position restraints or kcal/mol for dihedral angle restraints. mOR Wat referes to the crystal waters and seven additional water added using the program DOWSER.[1]

Supplementary Table 2 Steps in the equilibration simulation to relax the docked fentanyl-mOR complex structure

Step	Ensemble	Time (ns)	BB[a]	SC[a]	fentanyl[b]
1	NPT	1.00	2.50	1.25	2.50
2	NPT	1.00	1.25	0.75	1.25
3	NPT	1.00	1.00	0.50	1.00
4	NPT	1.00	0.50	0.25	0.50
5	NPT	1.00	0.25	0	0.25
6	NPT	110.0	0	0	0

[a] Force constant in the harmonic restraint potential for the backbone and sidechain heavy atoms in kcal/mol/Å^2. [b] Force constant in the harmonic restraint potential for the fentanyl heavy atoms.

Supplementary Table 3 Steps in the equilibration simulation to relax the H297 binding mode with H297 as either HIE or HIP

Step	Ensemble	Time (ns)	BB[a]	SC[a]	fentanyl[b]
1	NPT	1.00	2.50	1.25	2.50
2	NPT	1.00	1.25	0.75	1.25
3	NPT	1.00	1.00	0.50	1.00
4	NPT	1.00	0.50	0.25	0.50
5	NPT	1.00	0.25	0	0.25
6	NPT	60.0	0	0	0

[a] Force constant in the harmonic restraint potential for the backbone and sidechain heavy atoms in kcal/mol/Å^2. [b] Force constant in the harmonic restraint potential for the fentanyl heavy atoms.

Supplementary Table 4 Calculated pK_a's of mOR titratable residues and fentanyl from the pH replica-exchange CpHMD simulations

Residue	CpH-apo	CpH-D147	CpH-H297
Asp114	4.8	5.1	4.8
Asp147	3.3	4.1	3.8
Asp164	3.6	2.8	3.0
Asp177	3.6	3.8	3.8
Asp216	2.5	2.9	2.5
Asp272	2.6	2.0	1.9
Asp340	3.3	3.3	3.2
Glu229	3.2	2.8	2.9
Glu270	3.5	3.3	3.3
Glu310	3.8	3.2	3.7
Glu341	3.2	3.5	3.0
His54	6.8	7.3	7.5
His171	5.6	5.3	5.2
His223	5.8	5.8	5.7
His297	6.8	7.3	6.7
His319	7.1	7.2	6.8
Fentanyl	NA	>9.5[a]	>9.5[a]

[a] Only a lower bound to the pK_a of the piperidine amine of fentanyl is given, as the amine remains charged in the entire simulation pH range 2.5–9.5.

Supplementary Figures

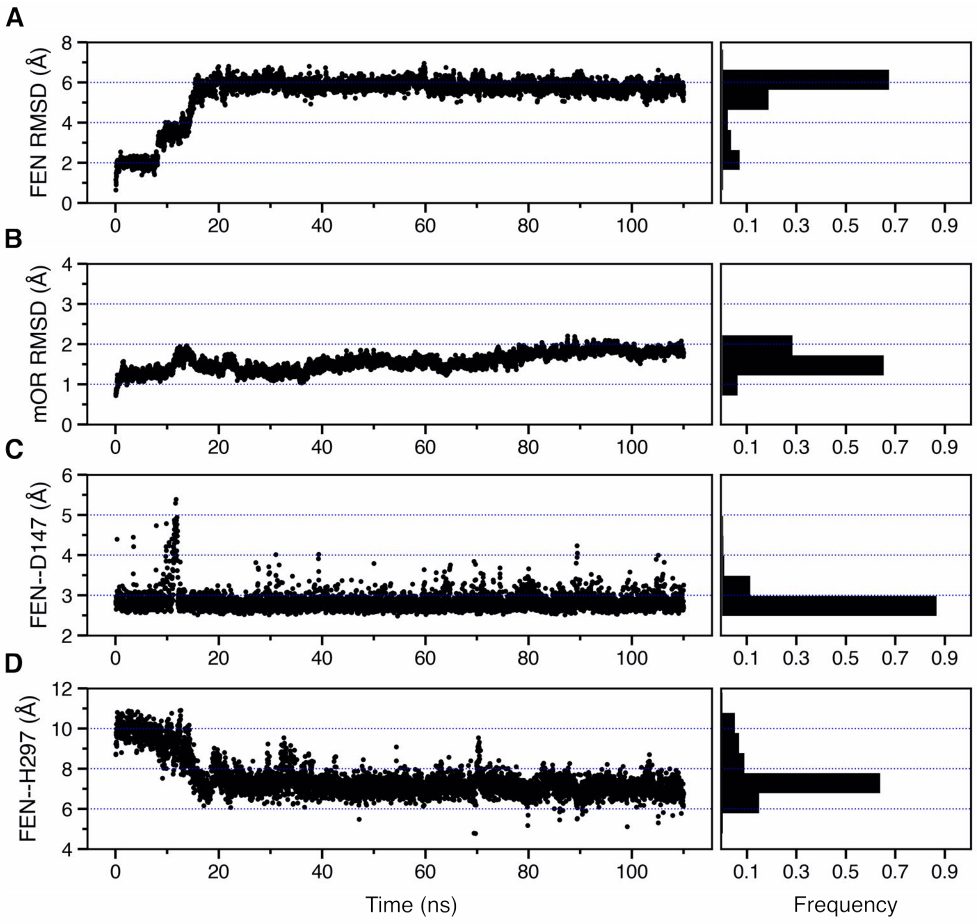

Supplementary Fig. 1 Equilibration of the docked fentanyl-mOR structure. A, B. Time series of the root-mean-square deviation (RMSD) of the fentanyl **(A)** and mOR **(B)** heavy atom positions with respect to their starting positions. **C.** Time series of the FEN–D147 distance, defined as the minimum distance between the piperidine nitrogen and the carboxylate oxygen of Asp147. **D.** Time series of the FEN–H297 distance, defined as that between the piperidine nitrogen and the unprotonated imidazole nitrogen of His297.

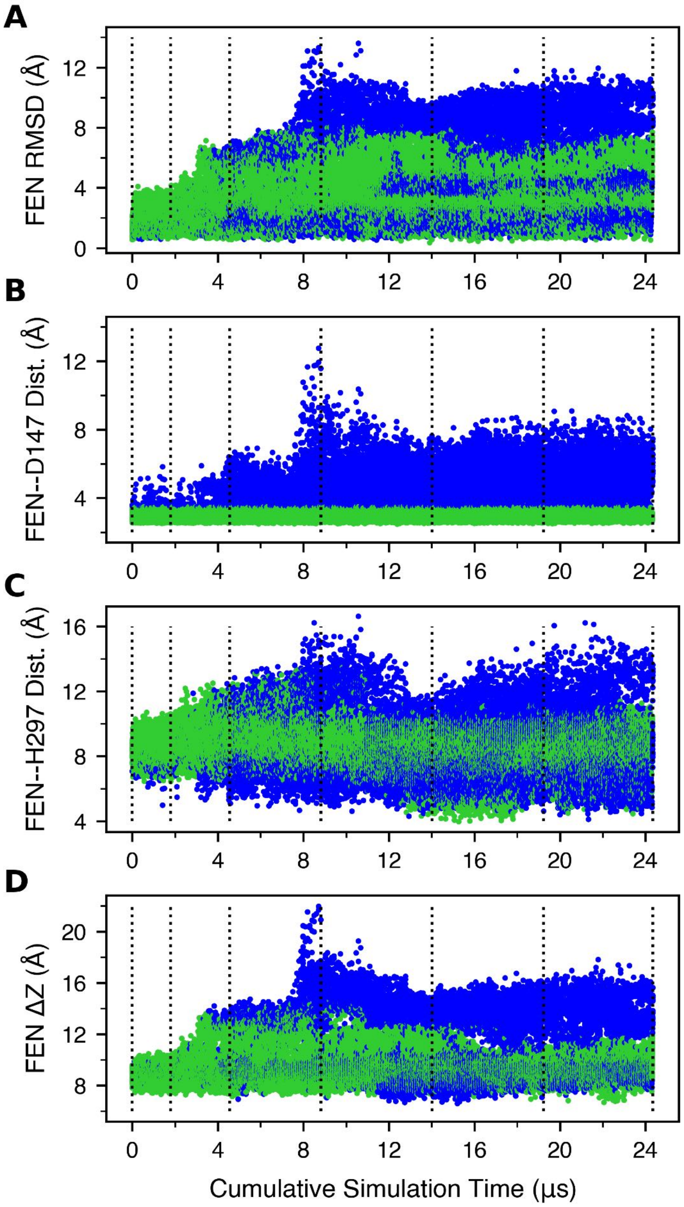

Supplementary Fig. 2 Fentanyl position as a function of the cumulative simulation time of the WE-HIE simulation. A. The root-mean-square deviation (RMSD) of the fentanyl heavy atoms with respect to the starting position. **B.** The FEN–D147 distance refers to the minimum distance between the piperidine nitrogen and the carboxylate oxygen of Asp147. **C.** The FEN–H297 distance is measured between the piperidine nitrogen and the unprotonated imidazole nitrogen of His297. **D.** Fentanyl ΔZ position is defined as the distance between the centers of mass (COM) of fentanyl and mOR in the z direction. Data with the FEN–D147 and FEN–H297 distances below 3.5 Å are colored green and orange, respectively, and otherwise blue. The unweighted data from all bins were taken and the time refers to the cumulative time. the vertical lines are drawn at every 50 weighted ensemble iterations.

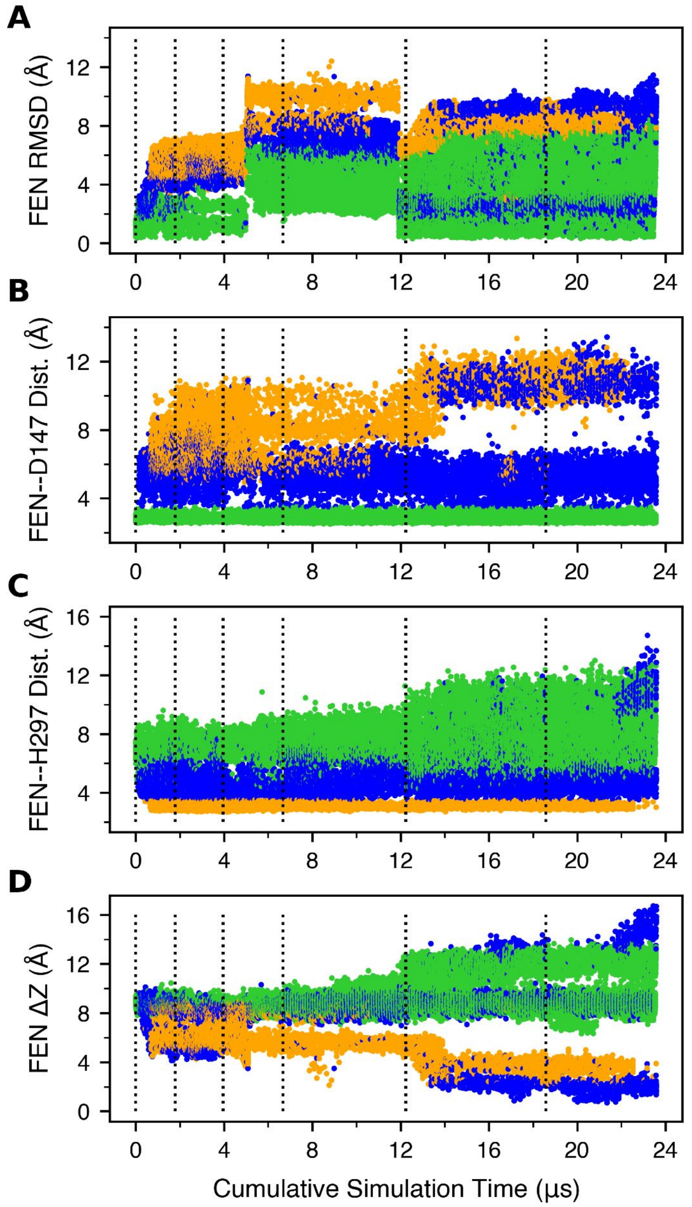

Supplementary Fig. 3 Fentanyl position as a function of the cumulative simulation time of the WE-HID simulation. **A.** The root-mean-square deviation (RMSD) of the fentanyl heavy atoms. An abrupt change near $\sim$5 μs is due to the manual bin boundary modification and does not affect the sampling results. **B.** The FEN–D147 distance refers to the minimum distance between the piperidine nitrogen and the carboxylate oxygen of Asp147. **C.** The FEN–H297 distance is measured between the piperidine nitrogen and the unprotonated imidazole nitrogen of His297. **D.** Fentanyl ΔZ position is defined as the distance between the centers of mass (COM) of fentanyl and mOR in the z direction. Data with the FEN–D147 and FEN–H297 distances below 3.5 Å are colored green and orange, respectively, and otherwise blue. The unweighted data from all bins were taken and the time refers to the cumulative time. the vertical lines are drawn at every 50 weighted ensemble iterations.

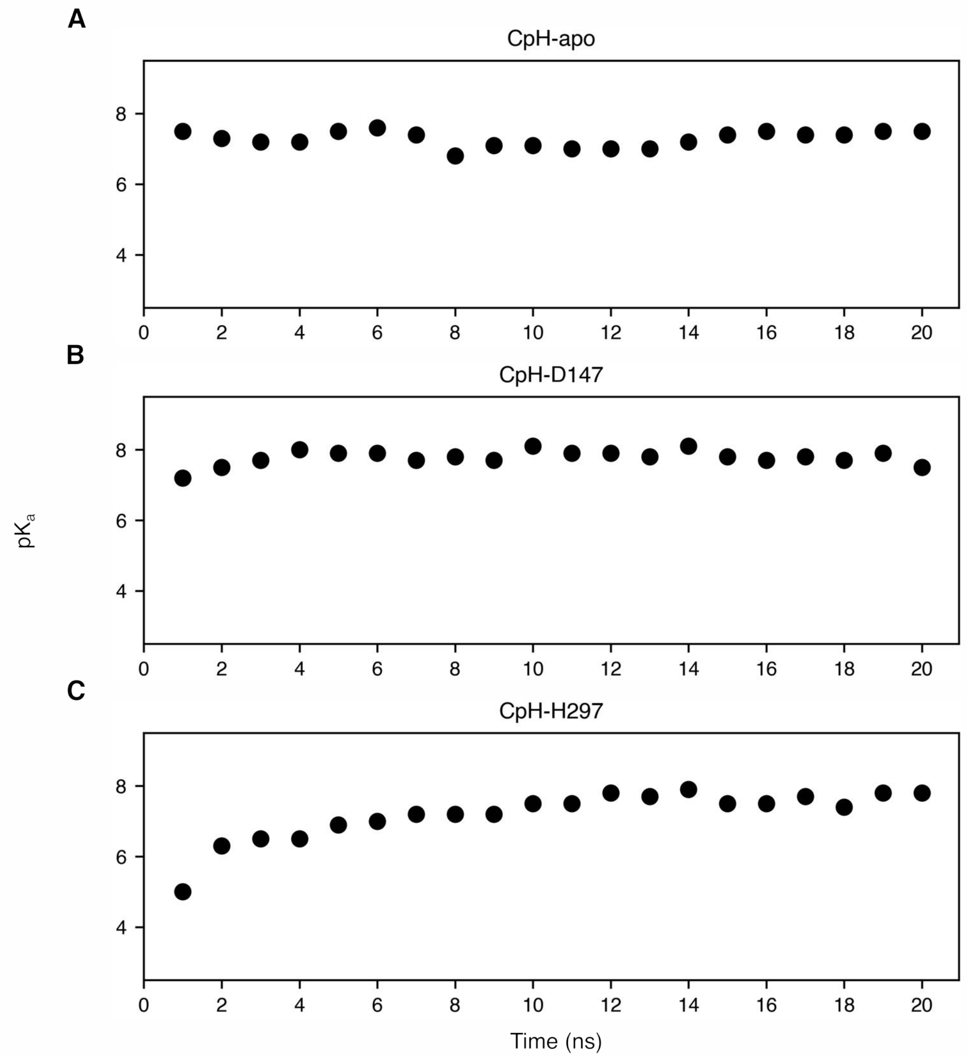

Supplementary Fig. 4 The pK_a of His297 is converged in all three sets of replica-exchange CpHMD simulations. The pK_a value of His297 plotted every 1-ns as a function of the single replica sampling time from the CpH-apo **(A)**, CpH-D147 **(B)**, and CpH-H297 **(C)** simulations.

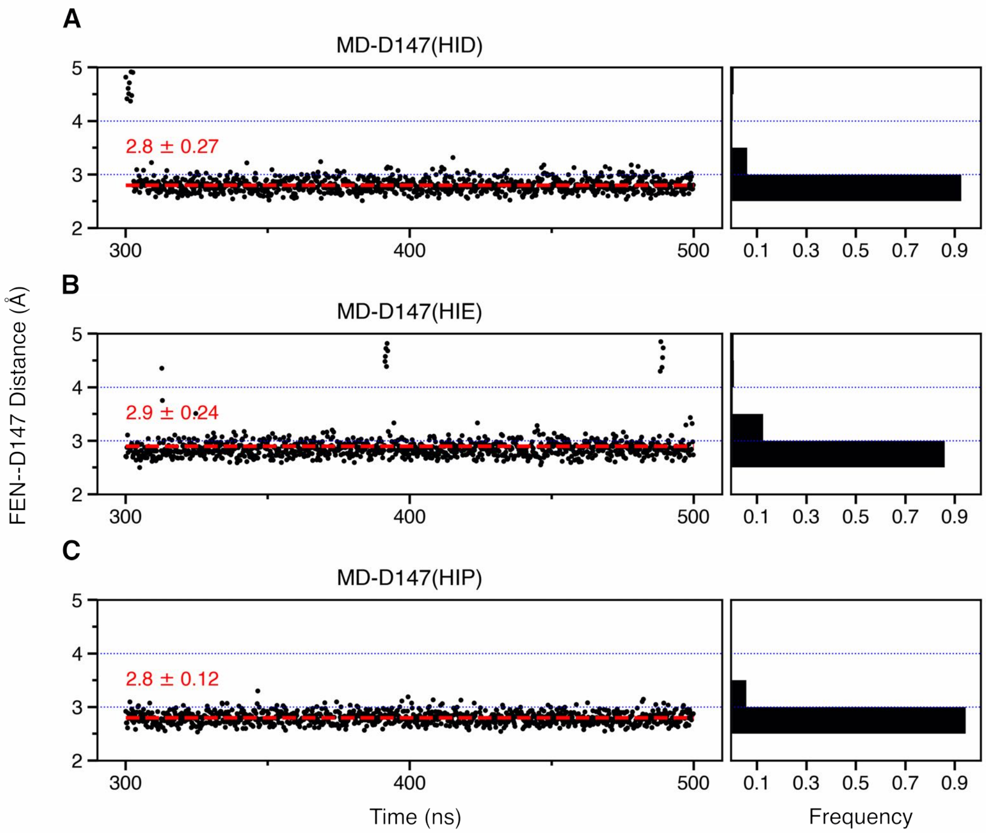

Supplementary Fig. 5 The D147 salt bridge is stable regardless of the His297 protonation state in the equilibrium simulations of the D147-binding mode. The time series of the FEN–D147 distance in the presence of HID297 **(A)**, HIE297 **(B)** , and HIP297 **(C)** in the equilibrium simulations of the D147-binding mode. The FEN–D147 distance refers to the minimum distance between the piperidine nitrogen and the carboxylate oxygen of Asp147.

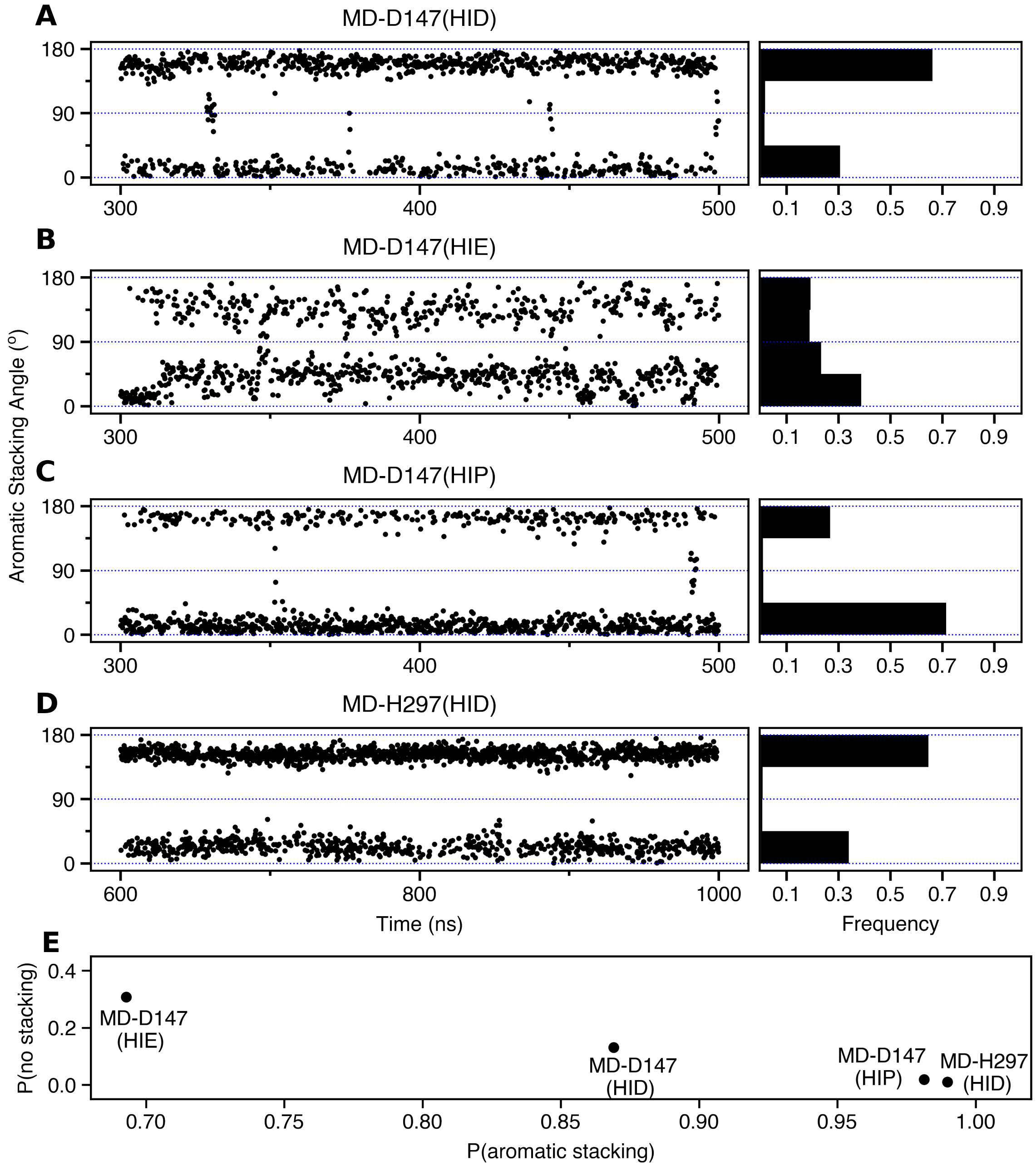

Supplementary Fig. 6 Aromatic stacking interaction between the phenyl ring of fentanyl's phenethyl group and Trp293 is persistent for both D147- and H297-binding modes. Time series of the fentanyl-W293 stacking angle from the D147-binding-mode simulations with HID297 **(A)**, HIE297 **(B)**, or HIP297 **(C)**, and from the H297-binding-mode simulation with HID297 **(D)**. **E.** Relationship between the probabilities of aromatic stacking and no aromatic stacking from each simulation. The angle is measured between the planes of the phenyl ring of fentanyl's phenethyl group and the six-member ring of Trp293. There is aromatic stacking interaction if the angle between the two planes is within 0–45° or 135–180°.

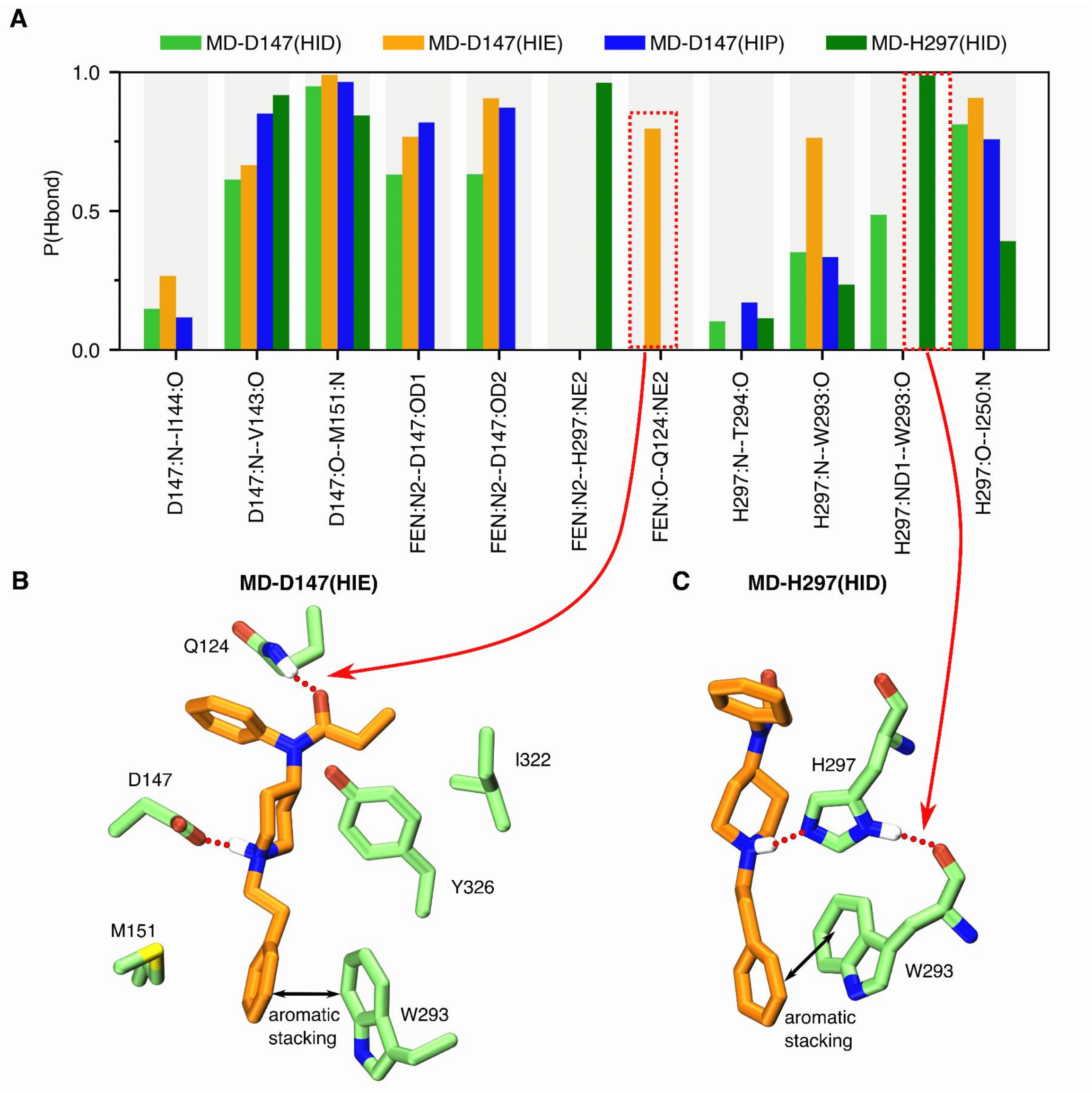

Supplementary Fig. 7 Occupancies of the intra- and intermolecular hydrogen bonds from the simulations of the D147- and H297-binding modes. The bars are colored as shown in top legend. A value of 1 means the hydrogen bond is persistent for the entire duration of the trajectory. A hydrogen bond is considered present if the donor-acceptor distance is below 3.5 Å and the acceptor-H-donor angle is less than 120°.

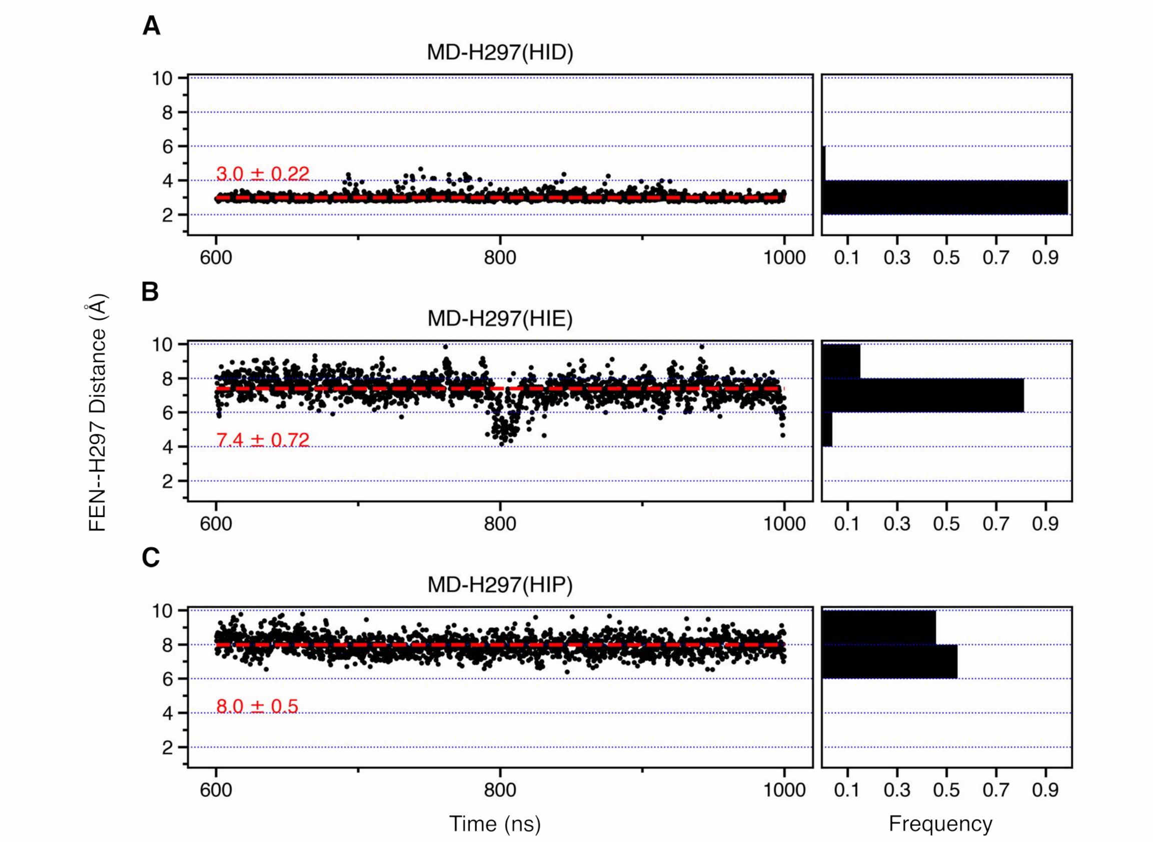

Supplementary Fig. 8 Fentanyl is pushed away from His297 in the equilibrium simulations of the H297-binding mode with HIE and HIP. The FEN–H297 distance as a function of simulation time in H297 mode with HID297 **(A)**, HIE297 **(B)**, and HIP297 **(C)**. The FEN–H297 distance is measured between the piperidine nitrogen and the unprotonated imidazole nitrogen of His297.

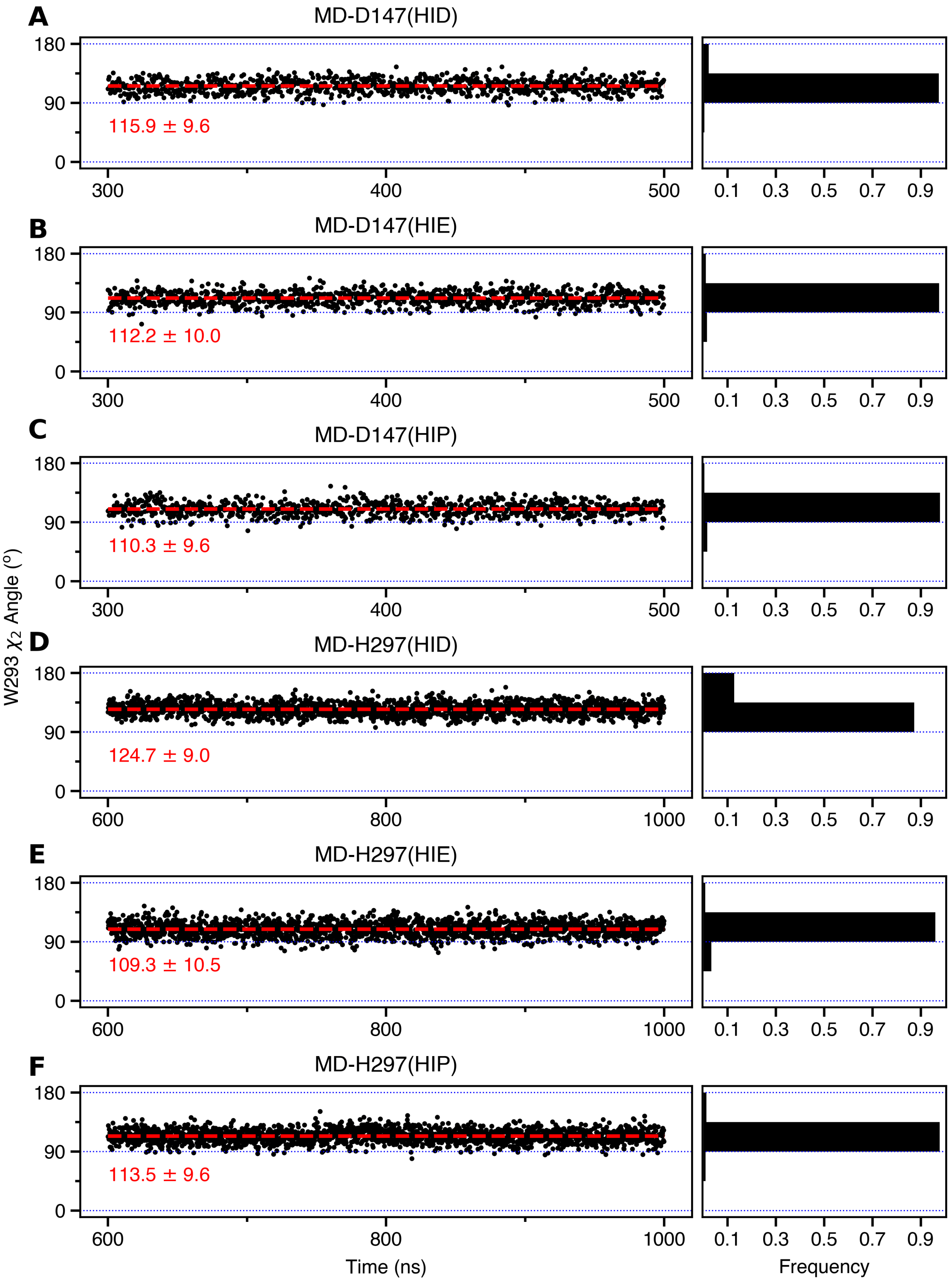

Supplementary Fig. 9 Trp293 χ_2 angle is somewhat decrease or increased relative to the value (120°) in the crystal structure of BU72-bound mOR (PDB 5C1M).[2] Time series of the Trp293 χ_2 angle in the simulations of the D147- (**A-C**) and the H297-binding mode (**D-F**).

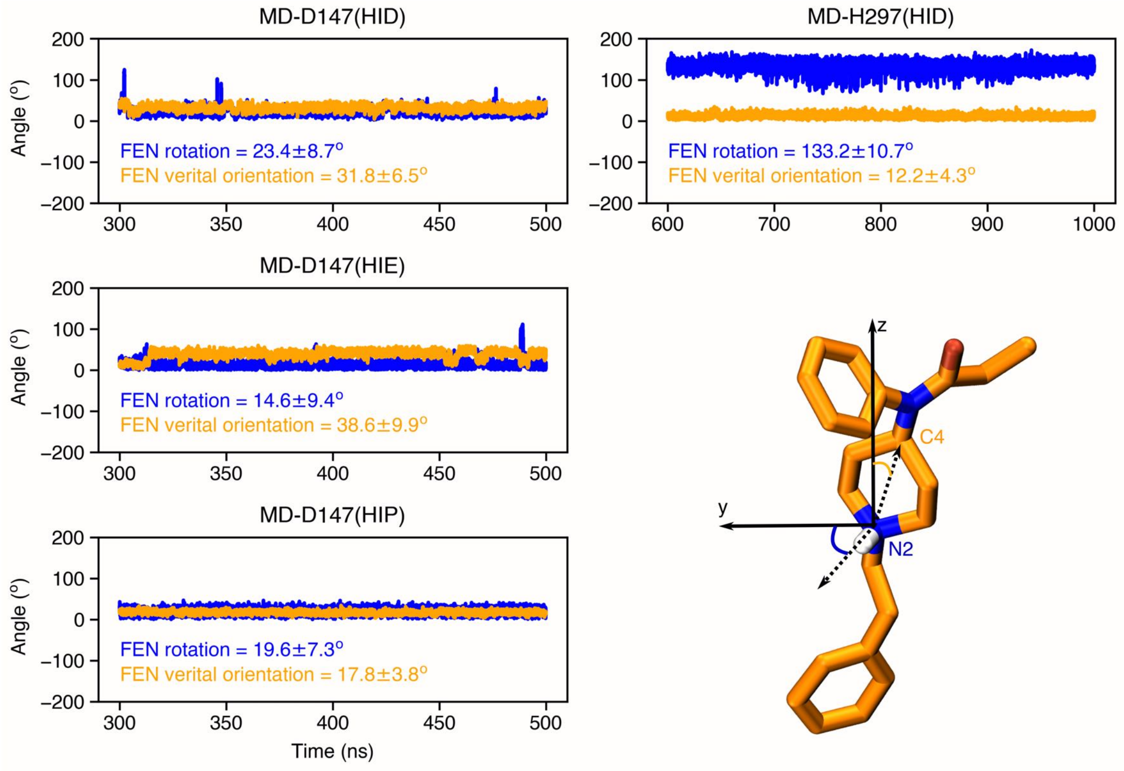

Supplementary Fig. 10 Fentanyl adopts different orientations in the simulations of the D147- and H297-binding modes. Time series of the fentanyl rotation angle (blue) and vertical orientation angle (gold). Fentanyl rotation angle is measured by the angle between N2–H vector and the y-axis. Fentanyl vertical orientation angle is measured by the angle between the N2–C4 vector and the membrane normal (z-axis).

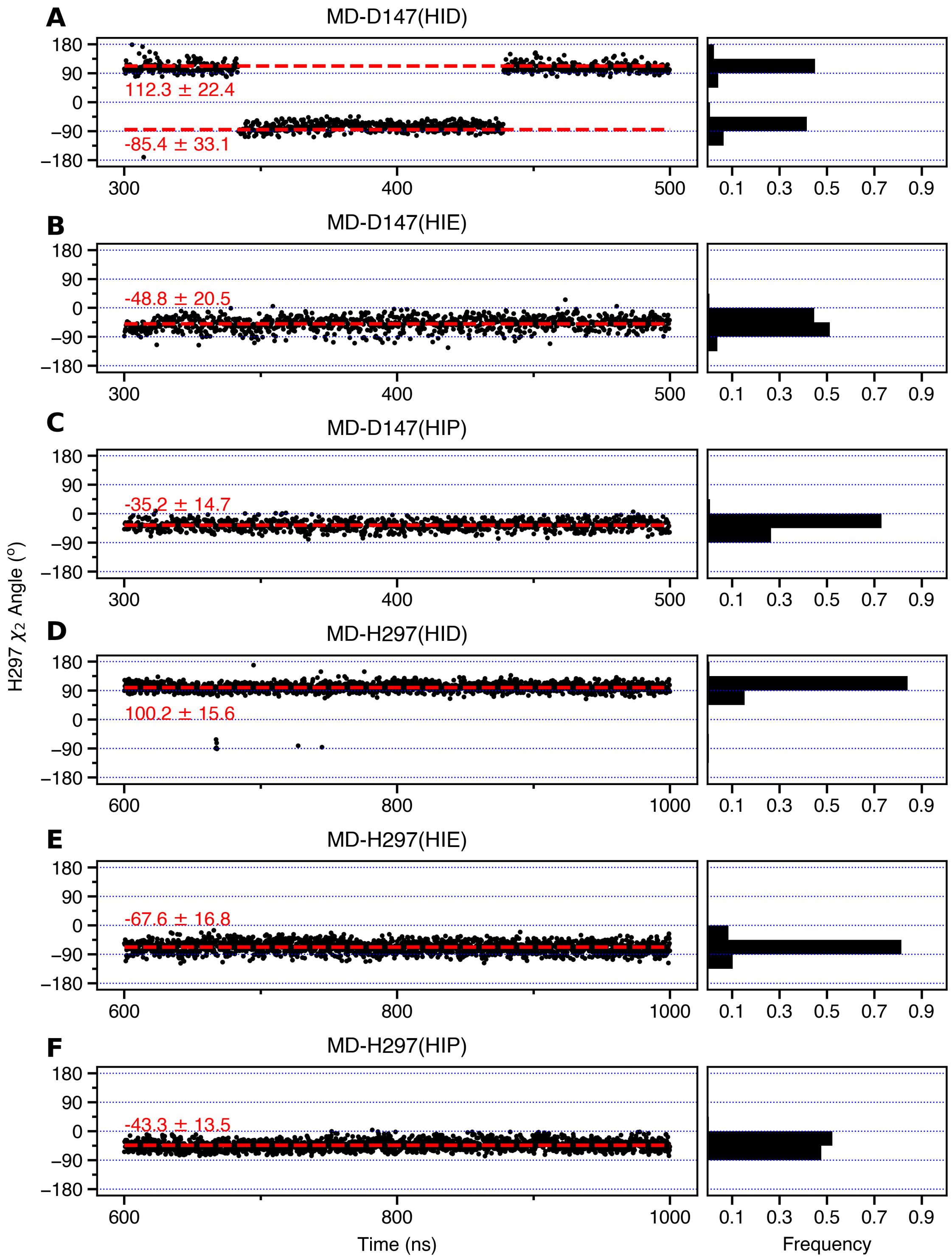

Supplementary Fig. 11 His297's χ_2 angle is influenced by the protonation/tautomer state. Time series of the χ_2 angle of His297 in the simulations of the D147-binding mode (**A-C**) and the H297-binding mode (**D-F**).

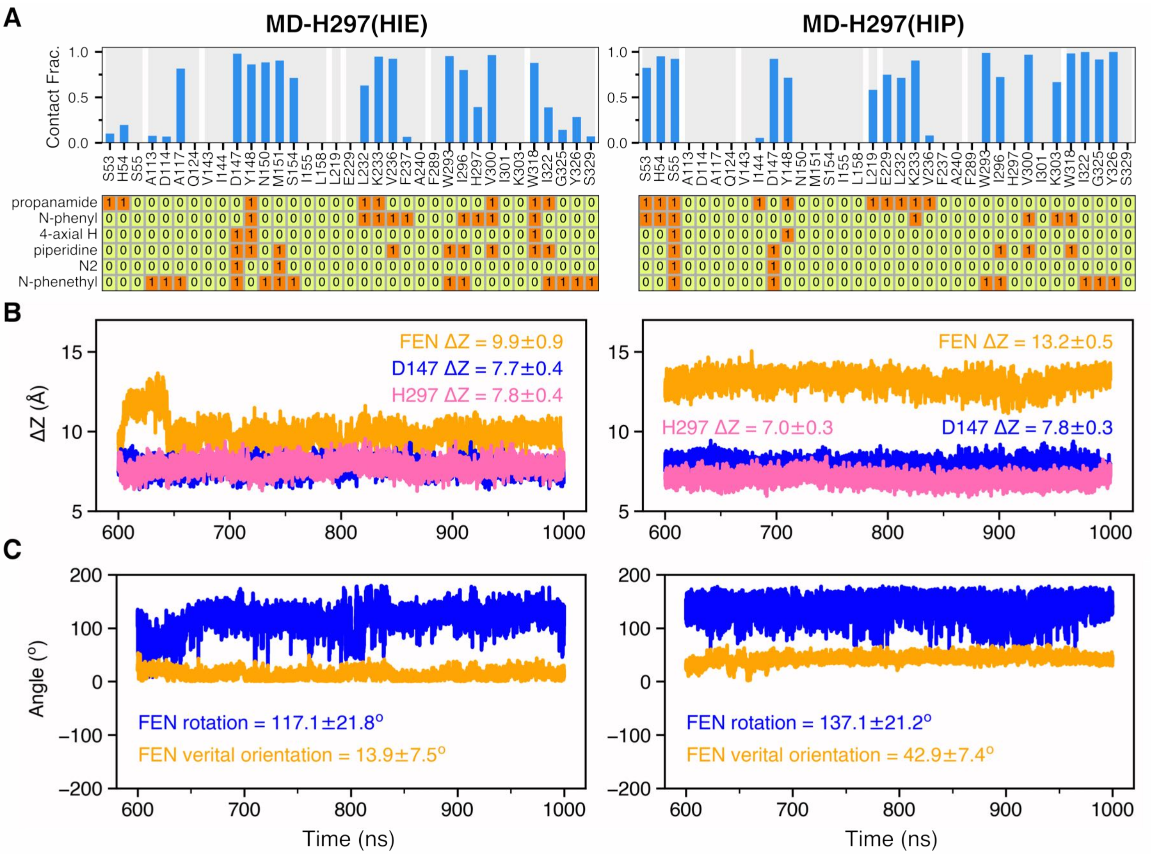

Supplementary Fig. 12 Characterization of the equilibrium simulations of the H297-binding mode with HIE297 and HIP297. MD-H297(HIE) (left column) and MD-H297(HIP) (right column) simulations. **A.** Fentanyl-mOR contact profile (top) and finger-print matrix (bottom) from the simulations with HIE297 (left) and HIP297 (right). Details are explained in the caption of Fig. 4 of the main text. **B.** Time series of the vertical positions of fentanyl (gold), Asp147 (blue), and His297 (magenta) from the simulations with HIE297 (left) and HIP297 (right). **C.** Time series of the fentanyl rotation angle (blue) and vertical orientation angle (gold) from the simulations with HIE297 (left) and HIP297 (right). Fentanyl rotation angle is measured by the angle between N2–H vector and the y-axis. Fentanyl vertical orientation angle is measured by the angle between the N2–C4 vector and the membrane normal (z-axis).

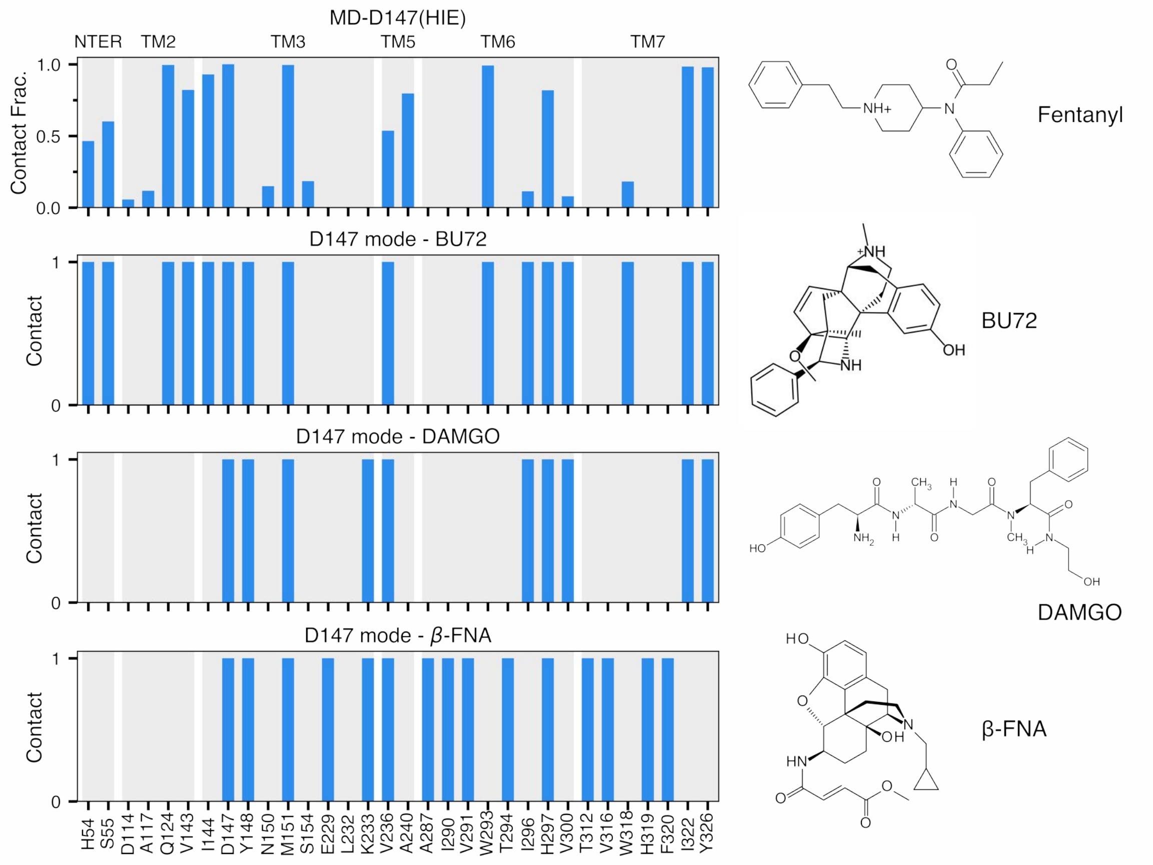

Supplementary Fig. 13 Comparison between fentanyl-mOR contact fraction to the BU72-, DAMGO-, β-FNA-mOR contacts in the crystal structures. MD-D147(HIE) was shown due to its highest T_c value compared to BU72 (Figure 5 in the main text). The N-terminal residues (residue 52 to 64) are not resolved in the crystal structures of DAMGO- and β-FNA-mOR.

References

(1) Zhang, L.; Hermans, J. Hydrophilicity of Cavities in Proteins. *Proteins* **1996**, *24*, 433.

(2) Huang, W. et al. Structural Insights into μ-Opioid Receptor Activation. *Nature* **2015**, *524*, 315–321.
